# Acceptability of and attitudes to the therapeutic use of cannabis and cannabidiol in people with Parkinson’s disease: A French survey

**DOI:** 10.1016/j.prdoa.2024.100286

**Published:** 2024-11-20

**Authors:** Tangui Barré, Géraldine Cazorla, Vincent Di Beo, Fabienne Lopez, Lise Radoszycki, Gwenaëlle Maradan, Christelle Baunez, Patrizia Carrieri

**Affiliations:** a Aix-Marseille Univ, Inserm, IRD, SESSTIM, Sciences Economiques & Sociales de la Santé & Traitement de l’Information Médicale, ISSPAM, Marseille, France; b Principes Actifs, Lieusaint, France; c Carenity, Paris, France; d ORS PACA, Southeastern Health Regional Observatory, Marseille, France; e Institut de Neurosciences de la Timone (INT) UMR7289, CNRS & Aix-Marseille Université, Marseille, France

**Keywords:** Cannabis, Cannabidiol, Acceptability, Anxiety, Symptoms, France

## Abstract

**Introduction:**

Cannabis and cannabidiol (CBD) may potentially alleviate symptoms and improve the quality of life of people with Parkinson’s disease (PD), although clinical results to date have provided conflicting evidence. In France, cannabis use is illegal outside the current restricted medical cannabis experimental framework which does not include PD as an eligible condition. In contrast, CBD products are legal and are easily available. We aimed to evaluate the acceptability of therapeutic cannabis and CBD use, and to assess cannabinoid-related attitudes among people with PD in France, with a view to assessing the potential inclusion of medical cannabinoids in PD treatment options.

**Methods:**

We conducted a French nationwide online survey among people with PD. Cannabis and CBD acceptability levels were derived from the answers to four questions. Logistic regressions were performed to identify factors associated with these levels. We also collected data on knowledge, information-seeking, and barriers to self-medication.

**Results:**

Of 1136 participants, acceptability levels of medical cannabis and CBD use were 81.7% and 87.4%, respectively. For both substances, acceptability was associated with the presence of anxiety symptoms, greater knowledge about cannabinoids, seeking information on medical cannabis, and considering the risk of cannabis dependence to be low. A fear of dependence was one of the main barriers to using either substance; healthcare providers were rarely mentioned as sources of information on medical cannabis.

**Conclusions:**

Acceptability levels of cannabis and CBD were high. Acceptability was associated with knowledge and perceptions of cannabinoids. Given ongoing misconceptions about the effects and risks associated with CBD, disseminating accurate information could increase its acceptability in people with PD.

## Highlights

• Cannabinoids may help people with PD to manage their symptoms.
• Acceptability of cannabis and cannabidiol was high in French people with PD.
• Acceptability was higher for cannabidiol than for cannabis.
• Fear of dependence is a strong barrier to the acceptability of cannabinoids for PD.

## 1 Introduction

People living with Parkinson’s disease (PD) have an impaired quality of life (QoL) in most domains [1] largely because of disease-associated symptoms [2], [3], [4]. One example is anxiety [5], [6], which can precede motor symptom onset, but may also be exacerbated by it [7]. Treatment side effects also contribute to impaired QoL in this population [3]. Long-term dopaminergic therapy for PD may lead to motor and non-motor complications which impair QoL [8], [9]. Poor response to levodopa is also common in late-stage PD. Moreover, the treatment options for non-motor symptoms − such as sleep disorders, depressive symptoms, and fatigue – are limited [8]. Deep brain stimulation is a surgical intervention that aims to improve the clinical state of PD patients; it has also been shown to improve patient QoL [10]. However, it is only available for patients who meet a certain number of criteria. Similarly, continuous subcutaneous apomorphine infusion, indicated in some patients who present motor fluctuations when taking oral medication, may improve PD patients’ QoL [11].

These various limitations highlight the importance of identifying alternative approaches to reduce disease and treatment burdens in PD patients. One possible approach is cannabis-based products. There are more than 550 chemical compounds in cannabis, with more than 100 phytocannabinoids identified [12]. Cannabinoids are molecules that interact with the endocannabinoid system, a lipid-based signaling system found throughout the human body. The most abundant and studied cannabinoids are tetrahydrocannabinol (THC) and cannabidiol (CBD). THC is psychoactive and responsible for the ‘high’ associated with cannabis use. On the contrary, CBD is well tolerated and non-intoxicating. Both molecules, whether used in combination or isolation in plant extracts or formulations, have multi-target effects on the human body, and may be beneficial in treating a wide range of ailments [13].

Although studies on PD in animal models have shown that cannabinoids may possibly help in alleviating motor symptoms [14], to date no study has provided compelling evidence that cannabinoid use is effective at reducing PD-related symptoms in patients [15], [16]. Nonetheless, previous reviews and a *meta*-analysis of clinical studies concluded that cannabinoids most likely constitute a safe option to potentially treat motor symptoms in PD; results for non-motor symptoms and QoL were even more conclusive [15], [17], [18]. However, a recent small randomized trial found no benefits of a cannabis extract treatment over a placebo on motor symptoms, sleep deterioration, cognition, or activities of daily living [19]. Studies based on real-world data have also highlighted that cannabis-based products may improve PD patients’ QoL [20], [21], [22], [23].

Patients’ attitudes towards medical cannabis may be shaped by their health status [24], and by social factors. The latter include cannabis-related stigma [25], [26], [27], [28], [29], [30] and exposure to different sources of information [31]. As cannabis is still primarily associated with recreational consumption, it may be difficult for persons with PD to consider that cannabis-based products may offer tangible therapeutic benefits.

In France, THC-containing products are illegal, except for an ongoing national medical cannabis experimental project initiated in March 2021, which aims to assess the feasibility of providing medical cannabis to individuals with chronic severe conditions not adequately alleviated by other treatments [32]. The project protocol stipulates that pharmacies can dispense cannabis-based products to persons holding a secure prescription from a trained specialist physician. Eligible indications are neuropathic pain, epilepsy, cancer symptoms, palliative situations, and painful spasticity. PD is currently not an eligible indication.

Unlike THC, and despite recent legal twists and turns, CBD is legal in France and is gaining popularity. Approximately 70 % of the French adult population have heard of it [33], and 10 % were users in 2021 and 2022 [33], [34]. CBD-based products are generally sold as wellness products or complimentary health products in France, in the form of oils, creams, THC-free cannabis flowers etc.

PD is already a qualifying condition for medical cannabis prescription in many US states [35]. It is possible that medical cannabis will become legal for PD in France in the future (whether as part of the current experimental project or over the longer term). In this context, the present study documents and analyzes French PD patients’ attitudes towards cannabis-based products and their acceptability of them, with a view to guiding future policymaking.

## 2 Material and methods

### 2.1 Study design and participants

We conducted the cross-sectional online survey CANNABAPA from 22 May to 14 July 2023. Inclusion criteria were being aged ≥ 18 years old and diagnosed with PD. Various sources displayed the link to the survey. One was the France Parkinson website (https://www.franceparkinson.fr/). Created in 1984 and a member of the European Parkinson’s Disease Association, France Parkinson is a national association recognized as being of public benefit; it has 75 local committees throughout France. Invitations to participate were sent by email to all contacts in the France Parkinson database (>35 000 addresses, including those of approximately 5000 association members). The survey link was also included in the France Parkinson newsletter, and published on its social media. Another source displaying the survey link was Carenity, a social network for people living with chronic conditions; it invited its members to participate through private messages on the network’s platform (1857 contacts, including relatives of PD patients). Furthermore, local associations of people living with PD promoted the survey. Finally, participants were encouraged to disseminate the link.

CANNABAPA was designed in accordance with the declaration of Helsinki, and was approved by the INSERM ethics committee (IRB00003888, CD/EB 23–045, 4 April 2023). It was powered by Voxco. Before accessing the survey questionnaire, participants had to provide informed consent.

### 2.2 Questionnaire and data collection

The self-administered online questionnaire collected data on socio-demographic characteristics including gender, age, type of area of residence (large city (defined as 200,000 inhabitants or more), medium-sized city/town, rural area), educational level, professional situation, and self-perceived household economic status.

Patients’ experience with their disease and treatment was measured using the following variables (i.e., questionnaire items): time since PD diagnosis, current intake of dopamine precursors and dopamine agonists (Yes/No), undergoing deep brain stimulation therapy (Yes/No), and two brief screening measures for anxiety and depression, specifically the Generalized Anxiety Disorder-2 (GAD-2) [36] and the Patient Health Questionnaire-2, (PHQ-2) [37]. The impact of fatigue was assessed with a question (‘Does fatigue or lack of energy limit your daytime activities?’, five possible answers) adapted from the Non-Motor Symptoms Scale for Parkinson’s Disease [38], [39], [40]. Disability level was assessed with the following item adapted from the Parkinson’s Disease Composite Scale: ‘To estimate your daily limitations, choose the statement that best corresponds to your current situation’ (six possible answers) [41], [42]. Pain was measured with three questions adapted from the Graded Chronic Pain Scale-Revised (frequency of pain, frequency of pain as a limiting factor in activities, and visual analog scale of average pain) [43]. Sleep quality was assessed with the following item: ‘During the past month, how would you rate your sleep quality overall?’ (four possible answers), taken from the Pittsburgh Sleep Quality Index [44].

Cannabinoid knowledge was assessed with four *ad hoc* statements each with three possible answers (True/False/Do not know) (see Supplementary Table 1), with an overall score ranging from 0 (no correct answer) to 4 (four correct answers). Self-information about medical cannabis was assessed with the question ‘Do you inform yourself about the medical use of cannabis for Parkinson’s disease?’ (three possible answers). Participants who responded ‘yes’ then reported between one and three primary sources of information, in their order of importance, from a list of pre-defined answers. Participant-perceived risk of dependence was assessed with the question ‘In your opinion, how great is the risk of becoming dependent on cannabis?’ (six possible answers). The participant’s position regarding the current legal status of cannabis in France was assessed with two questions: ‘Are you in favor of alleviating legal restrictions on medical [respectively, non-medical] use of cannabis in France?’ (three possible answers). Barriers to self-medication with cannabis and CBD were assessed with two questions: ‘What are the current barriers to your use of cannabis [respectively, CBD] as a self-medication for Parkinson’s disease?’. Participants could choose up to five barriers in order of importance (i.e., biggest to smallest barrier) from a pre-defined list of answers.

In the survey, cannabis was defined as containing THC above the French authorized level (0.3 %), and CBD was defined as any type of CBD-containing product with THC levels equal to or below the authorized level.

### 2.3 Study outcomes

The two study outcomes (i.e., acceptability of each of the two substances studied) were based on the following two questions: i) ‘Would you be inclined to use medical cannabis [respectively, CBD] (of controlled quality) for Parkinson's disease if it were prescribed to you?’; and ii) ‘Would you be inclined to use medical cannabis [respectively, CBD] (of controlled quality) for Parkinson's disease without prescription (i.e., over-the-counter)?’. Possible answers for each question were Yes/No/Do not know. Acceptability was classified as follows: ‘low’, for ‘No’ or ‘Do not know’ answers to both questions, ‘moderate’ for one ‘Yes’ answer, and ‘absolute’, when both answers were ‘Yes’.

### 2.4 Statistical analyses

We compared participants’ characteristics according to their cannabis and CBD acceptability level, using pairwise comparison (low vs. moderate, low vs. absolute, moderate vs. absolute). Chi-square and Mann-Whitney tests were performed for the qualitative and quantitative variables, respectively. Bonferroni corrections were applied to adjust for the higher risk of a type 1 error.

We then performed two separate binary logistic regression models, one for CBD and the other for cannabis, with a dichotomized moderate/absolute vs. low acceptability (reference category) status as the outcome. The following potential explanatory variables were included: socio-demographic characteristics, health-related variables, cannabinoid knowledge, self-information, and perceived risk of cannabis dependence. A p-value < 0.20 (Wald test) was the threshold value for eligible variables in the univariable analyses. A backward selection procedure was then used to obtain the two final multivariable models, with the p-value threshold for statistical significance set at 0.05.

Self-reported barriers to self-medication with cannabis-based products were presented separately for cannabis and CBD. A Chi-squared test was used to test for differences in the pattern of answers according to participants’ acceptability level. Self-reported primary sources of information were presented as percentages for participants who reported that they informed themselves about medical cannabis use. Stata/SE 16.1 software (StataCorp LP) was used for all analyses.

## 3 Results

### 3.1 Study sample characteristics

**Fig. 1:**
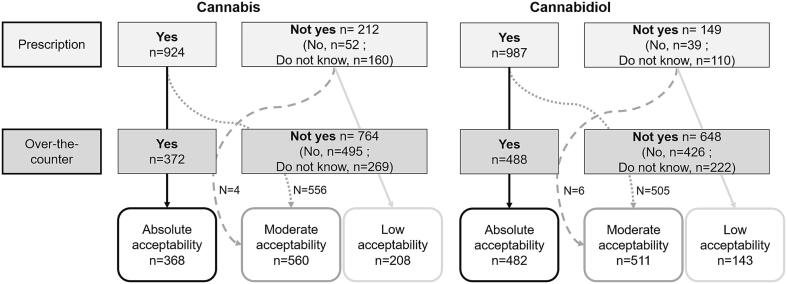
Classification of study participants according to their answers to the two outcome questions (n = 1136).

The survey was completed by 1136 participants (54.8 % men, median [interquartile range (IQR)] age 68 [62–74] years). Median [IQR] time since PD diagnosis was 7 [4.0–11.0] years. A third (34.6 %) of the study population experienced ‘limitations performing basic daily activities’ or a greater level of disability. The vast majority of participants (89.1 %) were directed to the survey through France Parkinson; 2.9 % were directed through Carenity. The respective distribution of participants according to cannabis and CBD acceptability level was as follows: 18.3 and 12.6 % low acceptability, 49.3 and 45.0 % moderate acceptability, and 32.4 and 42.4 % absolute acceptability (Fig. 1). The proportion of persons indicating absolute acceptability was significantly higher for CBD than for cannabis (proportion z-test, p < 0.001), and the proportion of persons indicating low acceptability was significantly higher for cannabis than for CBD (p < 0.001). The proportions of participants who answered ‘do not know’ to the prescription-based question (see above) were 14.1 % and 9.7 % for cannabis and CBD, respectively, while the proportions of participants answering ‘do not know’ to the non-prescription (i.e., over-the-counter) question were 23.7 % and 19.5 %. Study sample characteristics according to acceptability level are provided in Table 1.

**Table 1:** Study sample characteristics according to cannabis and cannabidiol acceptability.

**Characteristics (% of ‘do not know’)**	**All study sample** **N (%)**	**Low cannabis acceptability** **N (%)**	**Moderate cannabis acceptability** **N (%)**	**Absolute cannabis acceptability** **N (%)**	**Intergroup differences**^1^	**Low cannabidiol acceptability** **N (%)**	**Moderate cannabidiol acceptability** **N (%)**	**Absolute cannabidiol acceptability** **N (%)**	**Intergroup differences**^1^
**All study sample**	1136 (100)	208 (18.3)	560 (49.3)	368 (32.4)		143 (12.6)	511 (45.0)	482 (42.4)	
**Gender**									
Men	623 (54.8)	122 (58.7)	291 (52.0)	210 (57.1)		81 (56.6)	274 (53.6)	268 (55.6)	
Women	511 (45.0)	86 (41.3)	268 (47.9)	157 (42.7)		61 (42.7)	237 (46.4)	213 (44.2)	
Other	2 (0.2)	0 (0)	1 (0.2)	1 (0.3)		1 (0.7)	0 (0)	1 (0.2)	
**Age (in years, median [IQR])**	68 [62–74]	69 [62–74]	69 [62–74]	67 [60–74]		68 [62–73]	70 [63–75]	67 [60–73]	¤
**Area of residence**									
Rural area	376 (33.1)	59 (28.4)	195 (34.8)	122 (33.2)		42 (29.4)	175 (34.2)	159 (33.0)	
Medium-sized city	484 (42.6)	99 (47.6)	236 (42.1)	149 (40.5)		73 (51.0)	215 (42.1)	196 (40.7)	
Large city (>200 000 inhabitants)	276 (24.3)	50 (24.0)	129 (23.0)	97 (26.4)		28 (19.6)	121 (23.7)	127 (26.3)	
**Educational level**									§ ¤
< upper secondary school certificate	282 (24.8)	57 (27.4)	154 (27.5)	71 (19.3)		44 (30.8)	149 (29.2)	89 (18.5)	
upper secondary school certificate	166 (14.6)	34 (16.3)	80 (14.3)	52 (14.1)		23 (16.1)	74 (14.5)	69 (14.3)	
Tertiary educational diploma (< Master’s degree)	404 (35.6)	76 (36.5)	190 (33.9)	138 (37.5)		44 (30.8)	174 (34.1)	186 (38.6)	
Tertiary educational diploma (≥ >Master’s degree)	284 (25.0)	41 (19.7)	136 (24.3)	107 (29.1)		32 (22.4)	114 (22.3)	138 (28.6)	
**Professional situation**									¤
Retired	841 (74.0)	161 (77.4)	426 (76.1)	254 (69.0)		108 (75.5)	406 (79.5)	327 (67.8)	
Working	153 (13.5)	20 (9.6)	74 (13.2)	59 (16.0)		13 (9.1)	56 (11.0)	84 (17.4)	
Other (including occupational disability)	142 (12.5)	27 (13.0)	60 (10.7)	55 (14.9)		22 (15.4)	49 (9.6)	71 (14.7)	
**“Presently, would you say that in your household, financially speaking…?”**									
It’s difficult to make ends meet/You can’t manage without going into debt	110 (9.7)	23 (11.1)	46 (8.2)	41 (11.1)		21 (14.7)	40 (7.8)	49 (10.2)	
You just get by	265 (23.3)	52 (25.0)	128 (22.9)	85 (23.1)		33 (23.1)	127 (24.9)	105 (21.8)	
You are ok	445 (39.2)	85 (40.9)	230 (41.1)	130 (35.3)		59 (41.3)	197 (38.6)	189 (39.2)	
You are comfortable	316 (27.8)	48 (23.1)	156 (27.9)	112 (30.4)		30 (21.0)	147 (28.8)	139 (28.8)	
**Time since Parkinson’s disease diagnosis (in years, median [IQR]) (0.3)**	7 [4.0–11.0]	7.5 [4.0–11.5]	7.0 [4.0–11.0]	7.0 [4.0–11.0]		7.0 [4.0–12.0]	7.0 [4.0–12.0]	7.0 [4.0–11.0]	
**Taking dopamine precursors (0.6)**									
No	86 (7.6)	14 (6.8)	37 (6.6)	35 (9.6)		13 (9.2)	30 (5.9)	43 (9.0)	
Yes	1043 (92.4)	191 (93.2)	521 (93.4)	331 (90.4)		128 (90.8)	478 (94.1)	437 (91.0)	
**Taking dopamine agonists (3.0)**									
No	487 (44.2)	93 (45.1)	235 (43.8)	159 (44.2)		59 (41.8)	219 (44.8)	209 (44.3)	
Yes	615 (55.8)	113 (54.9)	301 (56.2)	201 (55.8)		82 (58.2)	270 (55.2)	263 (55.7)	
**Receiving deep brain stimulation**									
No	1046 (92.1)	189 (90.9)	515 (92.0)	342 (92.9)		130 (90.9)	467 (91.4)	449 (93.2)	
Yes	90 (7.9)	19 (9.1)	45 (8.0)	26 (7.1)		13 (9.1)	44 (8.6)	33 (6.8)	
**GAD-2 score**^3^**(median [IQR]) (7.0)**	2.0 [1.0–4.0]	2.0 [0.0–3.0]	2.0 [1.0–4.0]	2.0 [1.0–4.0]		1.0 [0.0–3.0]	2.0 [1.0–4.0]	2.0 [1.0–4.0]	# §
**GAD-2 score ≥ 3**^3^**(7.0)**									#
No	664 (62.9)	129 (68.6)	324 (62.4)	211 (60.5)		96 (73.3)	286 (60.7)	282 (62.1)	
Yes	392 (37.1)	59 (31.4)	195 (37.6)	138 (39.5)		35 (26.7)	185 (39.3)	172 (37.9)	
**PHQ-2 score (median [IQR])**^4^**(6.1)**	2.0 [0.0–3.0]	2.0 [0.0–3.0]	2.0 [0.0–3.0]	2.0 [1.0–3.0]		1.0 [0.0–3.0]	2.0 [0.0–3.0]	2.0 [1.0, 2.0]	
**PHQ-2 score ≥ 3**^4^**(6.1)**									
No	772 (72.4)	143 (74.5)	372 (70.7)	257 (73.6)		99 (74.4)	330 (69.0)	343 (75.2)	
Yes	295 (27.6)	49 (25.5)	154 (29.3)	92 (26.4)		34 (25.6)	148 (31.0)	113 (24.8)	
**Experiencing fatigue as a limit to daily activities**^5^									
Never / Rarely (< once/week)	220 (19.4)	40 (19.2)	104 (18.6)	76 (20.7)		33 (23.1)	87 (17.0)	100 (20.7)	
Regularly (once a week)	238 (21.0)	49 (23.6)	112 (20.0)	77 (20.9)		31 (21.7)	103 (20.2)	104 (21.6)	
Often (several times a week)	373 (32.8)	65 (31.3)	192 (34.3)	116 (31.5)		48 (33.6)	170 (33.3)	155 (32.2)	
Very often (every day)	305 (26.8)	54 (26.0)	152 (27.1)	99 (26.9)		31 (21.7)	151 (29.5)	123 (25.5)	
**Disability level**^6^									
Able to perform daily activity without problems.	297 (26.1)	52 (25.0)	150 (26.8)	95 (25.8)		34 (23.8)	130 (25.4)	133 (27.6)	
Limitations carrying out demanding daily activities or activities requiring fine motor skills.	446 (39.3)	83 (39.9)	218 (38.9)	145 (39.4)		63 (44.1)	190 (37.2)	193 (40.0)	
Limitations performing basic daily activities.	202 (17.8)	38 (18.3)	91 (16.3)	73 (19.8)		27 (18.9)	94 (18.4)	81 (16.8)	
Need help to perform some basic daily activities.	140 (12.3)	24 (11.5)	74 (13.2)	42 (11.4)		14 (9.8)	68 (13.3)	58 (12.0)	
Dependent on other persons to perform all basic daily activities.	51 (4.5)	11 (5.3)	27 (4.8)	13 (3.5)		5 (3.5)	29 (5.7)	17 (3.5)	
**Over the past three months, how often did you have pain?**^7^									
Never	118 (10.4)	21 (10.1)	49 (8.8)	48 (13.0)		13 (9.1)	48 (9.4)	57 (11.8)	
Some days	403 (35.5)	78 (37.5)	205 (36.6)	120 (32.6)		57 (39.9)	179 (35.0)	167 (34.6)	
Most days	267 (23.5)	49 (23.6)	132 (23.6)	86 (23.4)		30 (21.0)	129 (25.2)	108 (22.4)	
Every day	339 (29.8)	59 (28.4)	167 (29.8)	113 (30.7)		42 (29.4)	148 (29.0)	149 (30.9)	
Do not know	9 (0.8)	1 (0.5)	7 (1.3)	1 (0.3)		1 (0.7)	7 (1.4)	1 (0.2)	
**Over the past three months, how often did pain limit your life or work activities?**^7^									
Never	236 (20.8)	52 (25.0)	108 (19.3)	76 (20.7)		38 (26.6)	99 (19.4)	99 (20.5)	
Some days	492 (43.3)	95 (45.7)	241 (43.0)	156 (42.4)		61 (42.7)	219 (42.9)	212 (44.0)	
Most days	219 (19.3)	32 (15.4)	121 (21.6)	66 (17.9)		26 (18.2)	107 (20.9)	86 (17.8)	
Every day	169 (14.9)	25 (12.0)	80 (14.3)	64 (17.4)		14 (9.8)	78 (15.3)	77 (16.0)	
Do not know	20 (1.8)	4 (1.9)	10 (1.8)	6 (1.6)		4 (2.8)	8 (1.6)	8 (1.7)	
**Chronic pain**^8^**(1.1)**									
Absent	521 (46.4)	99 (48.1)	254 (46.0)	168 (45.9)		70 (49.6)	227 (45.0)	224 (46.8)	
Mild or bothersome	238 (21.2)	53 (25.7)	110 (19.9)	75 (20.5)		33 (23.4)	105 (20.8)	100 (20.9)	
High impact	365 (32.5)	54 (26.2)	188 (34.1)	123 (33.6)		38 (27.0)	172 (34.1)	155 (32.4)	
**Over the past three months, what number best describes your pain on average?**^7^**(median [IQR])**	5.0 [2.0–6.0]	5.0 [0.0–6.0]	5.0 [2.0–7.0]	4.0 [2.0–6.0]		5.0 [0.0–7.0]	5.0 [2.0–7.0]	4.0 [2.0–6.0]	
**During the past month, how would you rate** **your sleep quality overall?**^9^									
Very good	87 (7.7)	13 (6.3)	39 (7.0)	35 (9.5)		9 (6.3)	40 (7.8)	38 (7.9)	
Quite good	456 (40.1)	90 (43.3)	226 (40.4)	140 (38.0)		68 (47.6)	205 (40.1)	183 (38.0)	
Quite poor	441 (38.8)	79 (38.0)	225 (40.2)	137 (37.2)		46 (32.2)	206 (40.3)	189 (39.2)	
Very poor	152 (13.4)	26 (12.5)	70 (12.5)	56 (15.2)		20 (14.0)	60 (11.7)	72 (14.9)	
**Cannabinoid knowledge (median [IQR])**^10^	2.0 [1.0–3.0]	2.0 [0.0–3.0]	2.0 [1.0–3.0]	3.0 [2.0–4.0]	# § ¤	1.0 [0.0–2.0]	2.0 [1.0–3.0]	3.0 [2.0–4.0]	# § ¤
**Do you inform yourself about the medical use of cannabis for Parkinson’s disease?**					# § ¤				# § ¤
Not at all	409 (36.0)	117 (56.3)	232 (41.4)	60 (16.3)		96 (67.1)	214 (41.9)	99 (20.5)	
Yes, somewhat	509 (44.8)	68 (32.7)	251 (44.8)	190 (51.6)		36 (25.2)	232 (45.4)	241 (50.0)	
Yes, absolutely	218 (19.2)	23 (11.1)	77 (13.8)	118 (32.1)		11 (7.7)	65 (12.7)	142 (29.5)	
**In your opinion, how great is the risk of becoming dependent on cannabis?**					# § ¤				# § ¤
There is no risk	75 (6.6)	4 (1.9)	21 (3.8)	50 (13.6)		3 (2.1)	26 (5.1)	46 (9.5)	
Low risk	216 (19.0)	12 (5.8)	92 (16.4)	112 (30.4)		9 (6.3)	84 (16.4)	123 (25.5)	
Moderate risk	188 (16.5)	20 (9.6)	99 (17.7)	69 (18.8)		13 (9.1)	82 (16.0)	93 (19.3)	
High risk	289 (25.4)	74 (35.6)	143 (25.5)	72 (19.6)		48 (33.6)	123 (24.1)	118 (24.5)	
Very high risk	95 (8.4)	40 (19.2)	43 (7.7)	12 (3.3)		24 (16.8)	42 (8.2)	29 (6.0)	
Do not know	273 (24.0)	58 (27.9)	162 (28.9)	53 (14.4)		46 (32.2)	154 (30.1)	73 (15.1)	
**Are you in favor of alleviating legal restrictions on medical use of cannabis in France?**					# § ¤				# § ¤
No	81 (7.1)	46 (22.1)	27 (4.8)	8 (2.2)		39 (27.3)	28 (5.5)	14 (2.9)	
Yes	845 (74.4)	77 (37.0)	430 (76.8)	338 (91.8)		53 (37.1)	373 (73.0)	419 (86.9)	
Did not adopt a position	210 (18.5)	85 (40.9)	103 (18.4)	22 (6.0)		51 (35.7)	110 (21.5)	49 (10.2)	
**Are you in favor of alleviating legal restrictions on non-medical use of cannabis in France?**					# § ¤				# § ¤
No	373 (32.8)	92 (44.2)	199 (35.5)	82 (22.3)		64 (44.8)	185 (36.2)	124 (25.7)	
Yes	477 (42.0)	39 (18.8)	208 (37.1)	230 (62.5)		31 (21.7)	189 (37.0)	257 (53.3)	
Did not adopt a position	286 (25.2)	77 (37.0)	153 (27.3)	56 (15.2)		48 (33.6)	137 (26.8)	101 (21.0)	
**Cannabis acceptability status**									# § ¤
Low acceptability		−	−	−		126 (88.1)	43 (8.4)	39 (8.1)	
Moderate acceptability						15 (10.5)	441 (86.3)	104 (21.6)	
Absolute acceptability						2 (1.4)	27 (5.3)	339 (70.3)	

IQR, interquartile range; GAD, general anxiety disorder; PHQ, patient health questionnaire.

1 For a given substance (i.e., cannabis and cannabidiol), distribution differences between acceptability groups were tested with Chi-square test for categorical variables, and Mann-Whitney test for continuous variables, applying the Bonferroni correction for multiple comparison. The threshold of significance was set at 0.05. Only significant comparisons are displayed in the columns. # refers to low vs. moderate, § refers to low vs. absolute, and ¤ refers to moderate vs. absolute acceptability.

3 Generalized Anxiety Disorder scale-2, GAD-2 [36].

4 Patient Health Questionnaire, PHQ-2 [37].

5 Item adapted from the Non-Motor Symptoms Scale for Parkinson’s Disease [38], [39], [40].

6 Item adapted from the Parkinson’s Disease Composite Scale [41], [42].

7 Items adapted from the Graded Chronic Pain Scale-Revised [43].

8 Rated according to the two previous questions [43].

9 Item taken from the Pittsburgh Sleep Quality Index [44].

10 Scoring (0–4) based on correctly answering four *ad hoc* statements.

Across all three acceptability level groups, for both cannabis and CBD, differences were observed in cannabinoid knowledge, self-information, the perceived risk of cannabis dependence, and positions regarding the relaxation of current cannabis laws in France. Acceptability levels for cannabis and CBD were positively correlated (Table 1).

### 3.2 Factors associated with acceptability of medical cannabis and CBD.

The results of the multivariable analyses are provided in Table 2. For both cannabis and CBD, moderate/absolute acceptability levels were associated with a GAD-2 score ≥ 3, better cannabinoid knowledge, seeking information on medical cannabis, and considering the risk of cannabis dependence to be low (as compared to high/very high). Furthermore, CBD acceptability was associated with a better self-perceived household economic status.

**Table 2:** Factors associated with absolute/moderate (vs. low) cannabis and cannabidiol acceptability levels (n = 1136, multivariable logistic regression models).

	**Cannabis**		**Cannabidiol**	
	**aOR [95 % CI]**	**p-value**	**aOR [95 % CI]**	**p-value**
**“Presently, would you say that in your household, financially speaking…?”**				
It’s difficult to make ends meet/You can’t manage without going into debt	−	−	1	
You just get by / You are ok			2.10 [1.17–3.76]	0.012
You are comfortable			3.18 [1.63–6.22]	0.001
**GAD-2 score ≥ 3**^1^				
No	1		1	
Yes	1.48 [1.03–2.11]	0.033	1.92 [1.24–2.98]	0.003
Missing value (n = 80)	0.95 [0.54–1.69]	0.865	1.38 [0.69–2.77]	0.362
**Cannabinoid knowledge (median [IQR])**^2^	1.25 [1.09–1.43]	0.001	1.44 [1.22–1.69]	<0.001
**Do you inform yourself about the medical use of cannabis for Parkinson’s disease?**				
Not at all	1		1	
Yes, somewhat / absolutely	1.8 [1.27–2.55]	0.001	2.73 [1.80–4.14]	<0.001
**In your opinion, what is the risk of becoming dependent on cannabis?**				
There is no risk/a slight risk^3^	1		1	
Moderate risk	0.54 [0.27–1.08]	0.082	0.62 [0.27–1.42]	0.261
High/ Very high risk	0.16 [0.09–0.28]	<0.001	0.23 [0.12–0.44]	<0.001
Do not know	0.32 [0.17–0.59]	<0.001	0.38 [0.19–0.77]	0.007

aOR, adjusted odds ratio; CI, confidence interval.

1 Generalized Anxiety Disorder scale-2 [36].

2 Scoring (0–4) based on correctly answering four *ad hoc* statements.

3 ‘There is no’ and ‘slight’ were aggregated, as were ‘high’ and ‘very high’.

### 3.3 Self-reported barriers to self-medication with cannabis and CBD.

Supplementary Table 2 presents the reported barriers to self-medication with cannabis and CBD according to how frequently they were reported, and irrespective of their order of importance (see above). For cannabis, the most-cited barriers were ‘a lack of information about the right way to use it’ (42.4 %), ‘the fear of drug-drug interactions’ (42.2 %), and ‘the fear of other adverse effects’ (39.7 %). For CBD, the most-cited barriers were ‘a lack of information about the right way to use it’ (45.3 %), ‘the fear of drug-drug interactions’ (41.1 %), and ‘the absence of a recommendation from my physician’ (37.9 %).

**Fig. 2:**
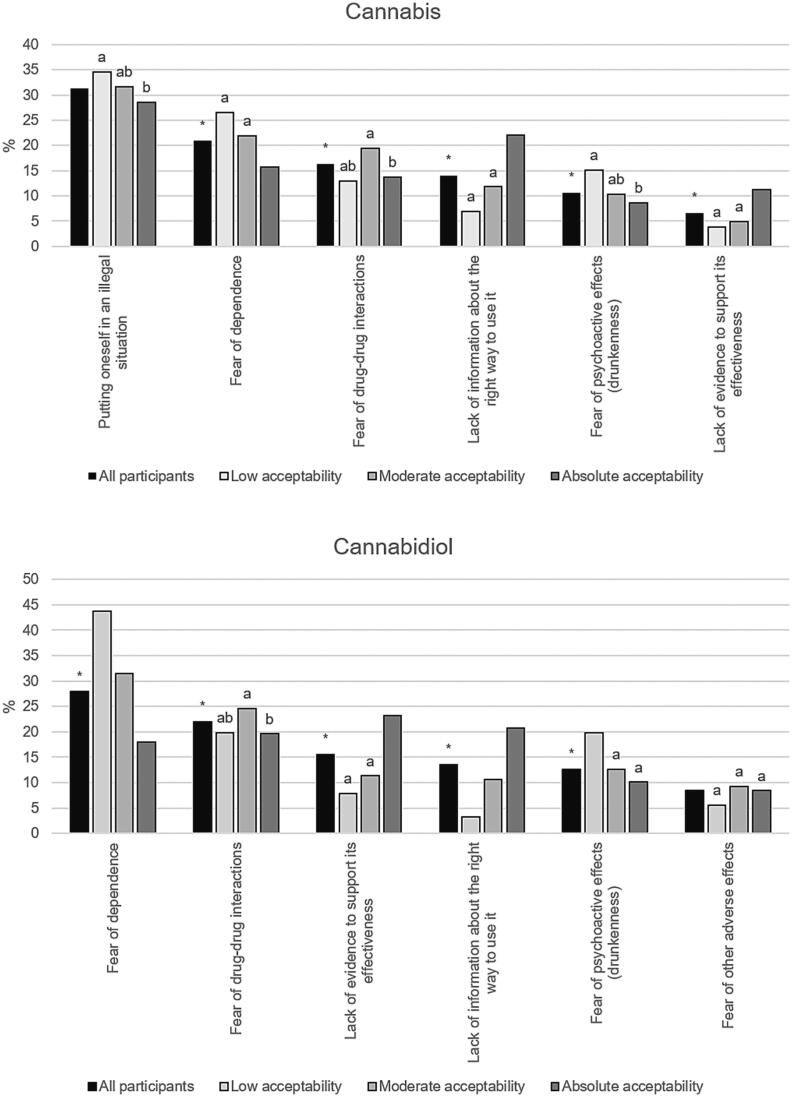
Percentages of cited primary barriers to self-medication for cannabis and cannabidiol according to acceptability level. Only barriers cited by more than 5 % of participants are shown. * indicates a global p-value < 0.05 (Chi-square test). For a given barrier, groups with a similar letter were not statistically different (p ≥ 0.05, Chi-square test). ‘Putting oneself in an illegal situation’ was not proposed as a possible answer for cannabidiol, as it is legal in France.

Barriers to self-medication reported as the primary (i.e., the biggest) barrier by > 5 % of the participants are provided in Fig. 2. For cannabis, the most cited primary barriers were ‘putting oneself in an illegal situation’, and ‘the fear of dependence’. The two most-cited primary barriers for CBD were ‘the fear of dependence’, and ‘the fear of drug-drug interactions’. Patterns of self-reported primary barriers differed according to acceptability level (Fig. 2).

### 3.4 Cannabinoid knowledge and self-reported sources of information

Of the four ad-hoc statements used to assess cannabinoid knowledge (see above), the highest rate of correct answers was for ‘Cannabidiol (CBD) is an active ingredient naturally present in the cannabis plant’ (73.5 %). The two statements on the abilities of CBD and THC to induce a high had high rates of ‘do not know’ answers (41.8 and 48.7 %, respectively). Finally, 24.3 % of the participants inaccurately thought that CBD was illegal (Supplementary Table 1).

Among the subgroup who reported seeking information about medical cannabis (n = 727), the most cited sources of information (irrespective of their ranking by respondent) were scientific and/or medical media (printed or online), and general media (including print press, television and internet sources). The two most cited primary sources of information (i.e., cited in first position by respondents) were scientific and/or medical media (printed or online), and patients and/or users on the internet (social media, forums etc.). Less than 30 % of participants cited their health professional as a source of information (Supplementary Table 3).

## 4 Discussion

This is the first study to investigate the factors associated with the acceptability of cannabis and CBD for medical purposes among patients with PD in France. Notably, both substances were deemed acceptable (i.e., moderate/absolute acceptability) by over 80 % of the participants. For both substances, acceptability was associated with a higher level of anxiety, actively seeking information for medical cannabis, greater knowledge of cannabinoids, and considering the risk of cannabis dependence to be low. Notably, knowledge gaps and the fear of dependence were very frequent barriers to patients choosing to self-medicate with either substance.

Previous studies on people living with PD investigating cannabis and/or CBD use focused on real-world prevalence of use [20], [22], [45], [46], [47], [48], [49]. Our acceptability measures were based on hypothetical situations (‘Would you be inclined to […] if […]’), and therefore provide a different kind of information from those studies. Accordingly, we cannot directly compare our findings with those from other studies. However, the 81.3 % and 86.8 % of participants who declared they would use, respectively, cannabis and CBD, if they were prescribed are similar to acceptability indicators in other settings and in patients with different medical conditions [50], [51], [52], [53], [54]. For example, more than 80 % of a preoperative patient cohort in the US reported that they would use cannabis if prescribed by a physician for pain after surgery or acute injury [55]. A study in the UK found that 86 % of participants with psychotic disorder were willing to try CBD as a treatment [56]. In the German general population, 48.3 % of CBD non-users declared they could imagine consuming CBD-containing products in the future [57].

We hypothesize that the high levels of acceptability for prescribed cannabis and CBD in our study were related to a high degree of patient trust in medical providers, although we have no data to support this. In a large quota-based sample of US individuals, Kurtzman et al. found that those who ‘completely’ trusted their usual clinician were more than twice as likely to report that they would definitely use medical cannabis if it were recommended [58].

In our study, 32.7 % and 43.0 % of participants declared they would use cannabis and CBD, respectively, without prescription (i.e., over-the-counter). Unlike the regulated use of prescription drugs, self-medication requires active engagement in self-management strategies and access to reliable information sources [59], [60]. In a US study involving a sample of patients with PD, only half reported using complementary or alternative medicine [22].

In our study, CBD use was much more acceptable than cannabis use. One possible reason is greater familiarity with the former, given the widespread distribution of specialized CBD shops in France. A second possible reason is that CBD is perceived as less harmful than cannabis. In a study of the French general population, fewer than 20 % of adults considered CBD to be ‘quite harmful’ or ‘very harmful’ [33]. We found that the fear of dependence and the fear of psychoactive effects as barriers to self-medication were more frequently cited for cannabis than for CBD.

Furthermore, after multiple adjustment, we found that the higher the perceived risk of dependence on cannabis, the lower the acceptability level for both cannabis and CBD. The fear of dependence was the second most frequently cited primary barrier to self-medication for cannabis, and the foremost barrier for CBD (though participants with higher levels of acceptability were significantly less likely to cite it). This vigilance and awareness of the risk of dependence may be related to dopaminergic treatments which can cause impulse control disorders in people with PD [61], [62]. PD patients with knowledge or experience of these treatments and their consequences may be particularly reluctant to use any substance linked to potential dependence.

Our findings concerning the perceived risk of dependence were similar for cannabis and CBD, which would suggest conflation of the characteristics of both substances in our sample of PD patients. Only 40.8 % of participants knew that CBD does not provoke a high, and 56.4 % that it is legal in France. In Germany, Yenilmez et al. reported that only 8.8 % of their sample of PD patients knew the difference between CBD and THC [46].

Better knowledge about cannabinoids was associated with greater willingness to use both cannabis and CBD in our study. Active information seeking was also associated with greater acceptability. Accordingly, providing accurate information about the potential of dependence on cannabinoids and their safety profile may enhance their acceptability.

Health professionals were rarely mentioned as a source of information on medical cannabis in our study; scientific and/or medical media, general media, and other patients and/or users on the internet were the principal sources cited. These two findings reflect results from a US study where the most common sources of information were from the internet/news and from friends or other people with PD [45]. In the Czech Republic, Venderová et al. reported that patients with PD mostly decided to take cannabis based on information presented in the media [47]. In Argentina, Micheli et al. reported that in their study sample of patients with PD, the main sources of information were friends, family or acquaintances, television, and the internet [49]. Our results regarding the principal sources also reflect findings in different populations, specifically older adults [63] and patients with multiple sclerosis [64].

All these findings highlight that in France, as elsewhere, in order to have reliable information on the medical use of cannabinoid, an individual needs to show active interest in the topic, and have the capacity to access and understand the information discovered. The fact that health professionals were cited so little as information sources in our study may suggest that patients were less likely to ask them for advice. Such reticence could be related to patients’ fear of disapproval by the provider, or explicit disapproval by physicians [65], [66], [67], [68] (including neurologists [69], [70]). Indeed, one can presuppose that given the lack of an approved cannabinoid-based product for PD in France, not all physicians may feel comfortable discussing this topic with their patients. As cannabis-based products cannot be prescribed by doctors, and pharmaceutical-grade CBD is not available over-the-counter in France, one cannot expect health professionals to initiate such a discussion or proactively provide information to patients.

Cannabis and CBD acceptability levels were only associated with one disease-related symptoms in the present study which was anxiety (positive association). Anxiety is very prevalent in people with PD [7], [71], although it is commonly underrecognized and undertreated [72]. Moreover, anxiety is a major independent predictor of QoL in PD patients [5], [7]. The associations we found between anxiety and acceptability levels may therefore be driven by both a strong unmet need for treatment to alleviate anxiety, and a perception that cannabis-based products are anxiolytic.

Clinical results to date for the benefits of CBD and cannabis in treating anxiety [73], [74], [75] are inconclusive. Both substances are commonly used in general populations for managing this condition [76], [77], [78], [79]. This is partly driven by as yet unproven claims transmitted through social media [80], [81], [82] and commercial marketing [83] that these products alleviate anxiety.

Participants in our study who perceived their household financial situation as difficult were more likely to have a low CBD acceptability level. One possible reason for this is that despite multiple adjustment for perceptions and knowledge, having financial difficulties was related to less favorable perceptions and/or less knowledge of CBD. In a large sample of older US adults, lower income was associated with higher perceived risks associated with cannabis consumption [84]. We can hypothesize that the same is true for CBD, given the highlighted conflation between both products. It has also been shown that lower income and lower socioeconomic status are associated with a lower likelihood of seeking online medical information [85], [86], [87], [88]. A second possible reason for lower acceptability in persons on a lower income is that the current prices of CBD products deterred them from buying them. Cost is a major issue for medical cannabis use in patients with chronic pain [89] and one can expect the same is true for CBD products. Prices for CBD products can be freely consulted by everybody in France.

This study has several strengths. First, it is the first to explore acceptability and attitudes to the therapeutic use of cannabis and CBD in PD patients in France. Second, the sample size was large, meaning good statistical power. Third, we assessed a complex mix of socio-demographic, health-related and perception-related variables, which allowed us to comprehensively characterize patients according to their acceptability level.

The study also has limitations. First, the sample cannot be considered representative of all French people with PD, and therefore we cannot generalize our results. More specifically, a very large percentage of the participants were directed to the survey from the national association France Parkinson. These people were therefore already engaged in information seeking and community-based exchanges. Accordingly, the relevant findings may be overestimated. Second, the web-based format of the survey may have disproportionally selected patients with fewer disabilities. However, one possible advantage of online surveying is reduced desirability bias, especially for a topic like cannabis-based products. However, it is likely that people interested in using cannabinoids were overrepresented, potentially leading to overestimations of acceptability. Third, the male-to-female ratio of our study sample was slightly lower than what would be expected in the national French PD population, as was the median age [90]. PD severity was only estimated, but not clinically validated. Finally, fatigue, sleep, and pain levels were assessed with isolated questionnaire items that do not reflect the psychometric properties of the validated scales they were taken from.

## 5 Conclusions

In conclusion, acceptability levels among people with PD for cannabis and CBD use were high, the latter substance being more acceptable. Our findings underscore that knowledge and perceptions of cannabinoids had a major impact on acceptability levels. As misconceptions persist about the negative effects of CBD and the risk of dependence, disseminating accurate information should increase acceptability.

## CRediT authorship contribution statement

**Tangui Barré:** Writing – original draft, Methodology, Conceptualization. **Géraldine Cazorla:** Writing – review & editing, Methodology, Conceptualization. **Vincent Di Beo:** Writing – review & editing, Formal analysis. **Fabienne Lopez:** Writing – review & editing, Methodology. **Lise Radoszycki:** Writing – review & editing, Investigation. **Gwenaëlle Maradan:** Writing – review & editing, Investigation. **Christelle Baunez:** Writing – review & editing, Conceptualization. **Patrizia Carrieri:** Writing – review & editing, Methodology, Conceptualization.

## Declaration of competing interest

The authors declare that they have no known competing financial interests or personal relationships that could have appeared to influence the work reported in this paper.
