# Supplementary material for: Acceptability of and attitudes to the therapeutic use of cannabis and cannabidiol in people with Parkinson’s disease: A French survey
Source: Clin Park Relat Disord. 2024 Nov 20;11:100286. doi: 10.1016/j.prdoa.2024.100286 (PMC11647636; doi:10.1016/j.prdoa.2024.100286)
Supplement: Supplementary Data 1 [file mmc1.docx]

**Supplementary Table 1: *Ad hoc* statements assessing knowledge about cannabinoids, according to participants’ answers (n=1136)**

|  | True | False | Do not know |
| --- | --- | --- | --- |
|  | **n (row %)** | **n (row %)** | **n (row %)** |
| Cannabidiol (CBD) is an active ingredient naturally present in the cannabis plant | 835 (73.5) | 13 (1.1) | 288 (25.4) |
| Cannabidiol (CBD) can impair some mental abilities (i.e., it can induce a *high*) | 197 (17.3) | 464 (40.8) | 475 (41.8) |
| The active ingredient tetrahydrocannabinol (THC) can impair some mental abilities (i.e., it can induce a *high*) | 464 (40.8) | 119 (10.5) | 553 (48.7) |
| Cannabidiol (CBD) is illegal in France | 276 (24.3) | 641 (56.4) | 219 (19.3) |

Correct answers are underlined.

**Supplementary Table 2. Cited barriers to self-medication, irrespective of their order of importance (n=1136)**

|  |  | **Cannabis acceptability** | | | |  | **Cannabidiol acceptability** | | | |
| --- | --- | --- | --- | --- | --- | --- | --- | --- | --- | --- |
| **%** | **All** | **Low** | **Moderate** | **Absolute** | **p-value^1^** | **All** | **Low** | **Moderate** | **Absolute** | **p-value^1^** |
| Lack of information about the right way to use it | 42.4 | 28.8 | 45.2 | 45.9 | <0.001 | 45.3 | 27.3 | 48.3 | 47.5 | <0.001 |
| Fear of drug-drug interactions | 42.2 | 49.5 | 47.1 | 30.4 | <0.001 | 41.1 | 49.7 | 49.7 | 29.5 | <0.001 |
| Fear of other adverse effects | 39.7 | 56.7 | 42.1 | 26.4 | <0.001 | 36.8 | 51.7 | 43.6 | 25.1 | <0.001 |
| Fear of dependence | 36.1* | 53.8 | 38.2 | 22.8 | <0.001 | 26.8 | 48.3 | 31.5 | 15.6 | <0.001 |
| Fear of psychoactive effects (*high*) | 35.9* | 57.2 | 37.0 | 22.3 | <0.001 | 25.3 | 44.8 | 30.3 | 14.1 | <0.001 |
| The absence of a recommendation from my physician | 32.7* | 27.9 | 39.5 | 25.0 | <0.001 | 37.9 | 36.4 | 48.7 | 27.0 | <0.001 |
| Putting oneself in an illegal situation | 31.3 | 37.5 | 32.1 | 26.6 | 0.022 | - |  |  |  |  |
| The unsecured quality of products | 28.3* | 16.8 | 28.2 | 34.8 | <0.001 | 23.8 | 11.2 | 24.9 | 26.3 | 0.001 |
| Lack of evidence to support its effectiveness | 22.8* | 21.6 | 22.5 | 23.9 | 0.799 | 29.4 | 30.8 | 27.4 | 31.1 | 0.406 |
| Its price | 19.5* | 11.5 | 16.1 | 29.1 | <0.001 | 23.2 | 8.4 | 21.1 | 29.9 | <0.001 |
| Difficulties in supply | 13.6* | 3.8 | 10.5 | 23.6 | <0.001 | 7.3 | 4.9 | 5.3 | 10.2 | 0.006 |
| My physician’s reluctance | 9.2 | 7.7 | 10.2 | 8.4 | 0.478 | 9.9 | 12.6 | 11.4 | 7.5 | 0.062 |
| Its form/way of administration is poorly adapted to my abilities | 3.3 | 2.9 | 3.2 | 3.8 | 0.816 | 3.1 | 2.8 | 2.5 | 3.7 | 0.543 |
| My relatives' reluctance | 1.7 | 1.4 | 1.8 | 1.6 | 0.944 | 1.5 | 2.8 | 1.2 | 1.5 | 0.366 |
| Fear of stigmatization (social disapproval) | 0.6 | 0.5 | 0.4 | 1.1 | 0.366 | 1.4 | 2.8 | 1.4 | 1.0 | 0.291 |

**^1^** Chi-square test

* Significant difference proportion for cannabis vs. cannabidiol (proportion z-test; p<0.05)

**Supplementary Table 3. Sources of information on medical cannabis among those who reported seeking information (n=727)**

|  | **Cited as primary source** | **Cited as a source** |
| --- | --- | --- |
|  | **n (%)** | **n (%)** |
| Scientific and/or medical media (printed or online) | 268 (36.9) | 394 (54.2) |
| Patients and/or users on the internet (social media, forums etc.) | 163 (22.4) | 202 (27.8) |
| General media (printed press, television, internet etc.) | 107 (14.7) | 294 (40.4) |
| Acquaintances (excluding patients/users or medical professionals) | 73 (10.0) | 245 (33.7) |
| My health professionals | 66 (9.1) | 205 (28.2) |
| Patients and/or users through other means (face-to-face, telephone communication) | 40 (5.5) | 68 (9.4) |
| Other | 10 (1.1) | 38 (5.2) |
